# Preclinical Activity of Simvastatin Induces Cell Cycle Arrest in G1 via Blockade of Cyclin D-Cdk4 Expression in Non-Small Cell Lung Cancer (NSCLC)

**DOI:** 10.3390/ijms14035806

**Published:** 2013-03-12

**Authors:** Yu-Wei Liang, Chi-Chang Chang, Chao-Ming Hung, Tzu-Yu Chen, Tzuu-Yuan Huang, Yi-Chiang Hsu

**Affiliations:** 1 Department of Emergency Medicine, Chi-Shan Hospital, Department of Health, Executive Yuan, Kaohsiung 84274, Taiwan; E-Mail: viclyan@yahoo.com.tw; 2 Department of Obstetrics & Gynecology, E-Da Hospital, E-Da Hospital/I-Shou University, Kaohsiung 82445, Taiwan; E-Mail: ed101779@edah.org.tw; 3 Department of General Surgery, E-Da Hospital, I-Shou University, Kaohsiung 82445, Taiwan; E-Mail: ed100647@edah.org.tw; 4 Innovative Research Center of Medicine, College of Health Sciences, Chang Jung Christian University, Tainan 71101, Taiwan; E-Mail: peggy811030@yahoo.com.tw; 5 Department of Bioscience Technology, College of Health Sciences, Chang Jung Christian University, Tainan 71101, Taiwan; 6 Departments of Neurosurgery, Tainan Sin-Lau Hospital, Tainan 70142, Taiwan; E-Mail: slh3353@sinlau.org.tw; 7 Graduate Institute of Medical Science, College of Health Sciences, Chang Jung Christian University, Tainan 71101, Taiwan

**Keywords:** simvastatin, non-small cell lung cancer, cell cycle arrest, CyclinD-Cdk4

## Abstract

Lung cancer is the most common cause of cancer-related death. Nonetheless, a decrease in overall incidence and mortality has been observed in the last 30 years due to prevention strategies and improvements in the use of chemotherapeutic agents. In recent studies, Simvastatin (SIM) has demonstrated anti-tumor activity, as well as potent chemopreventive action. As an inhibitor of 3-hydroxy-3-methylglutaryl-coenzyme A reductase (HMG-CoA), SIM has been shown to stimulate apoptotic cell death. In this study, an MTT assay revealed the cytotoxic activity of SIM against human large cell lung cancer (Non-small cell lung cancer; NSCLC) cells (NCI-H460); however, induced apoptosis was not observed in NCI-H460 cells. Protein expression levels of cell cycle regulating proteins Cdk4, Cyclin D1, p16 and p27 were markedly altered by SIM. Collectively, our results indicate that SIM inhibits cell proliferation and arrests NCI-H460 cell cycle progression via inhibition of cyclin-dependent kinases and cyclins and the enhancement of CDK inhibitors p16 and p27. Our findings suggest that, in addition to the known effects on hypercholesterolemia therapy, SIM may also provide antitumor activity in established NSCLC.

## 1. Introduction

Lung cancer is the leading cause of cancer-related death worldwide. The survival rate of lung carcinoma has remained relatively unchanged below 15% for the past two decades [1]. NSCLC accounts for approximately 80% of all forms of lung cancer, and early-stage NSCLC is potentially curative through complete surgical resection. Nonetheless, the overall five-year survival rate for these patients is only 45% to 70%, due to metastatic recurrence [2].

Statins have been shown to alter several cellular mechanisms, resulting in apoptosis and reduced tumor cell growth, as well as angiogenesis and impaired metastatic processes [3]. Simvastatin (SIM), like statins, is used in hypercholesterolemia therapy to inhibit the 3-hydroxy-3-methylglutarylcoenzyme A (HMG-CoA) reductase, which catalyzes the conversion of HMG-CoA to mevalonate [4]. In addition to its cholesterol-lowering activity, evidence is mounting to support claims that SIM may have potential anti-cancer benefits [5,6]. SIM appears to mediate angiogenesis, cell proliferation, distant metastasis, cell cycle arrest and apoptosis, as well as suppress NF-κB activation [7,8]. The anti-tumor effects of SIM have been demonstrated in a variety of tumors, including myeloma cells [9], breast cancer [10], leukemia [11], prostate cancer [12], malignant glioblastoma [13] and hepatoma [14]; however, the anti-tumor effects of SIM on NSCLC have not been studied in depth.

This study characterized the anti-proliferation, cell cycle arrest and anti-tumorigenesis activities of Simvastatin on NCI-H460 cells. Our results provide valuable data for the further development of treatments for NSCLC.

## 2. Results and Discussion

### 2.1. SIM Inhibits the Proliferation of NCI-H460 Cells

**Figure 1:**
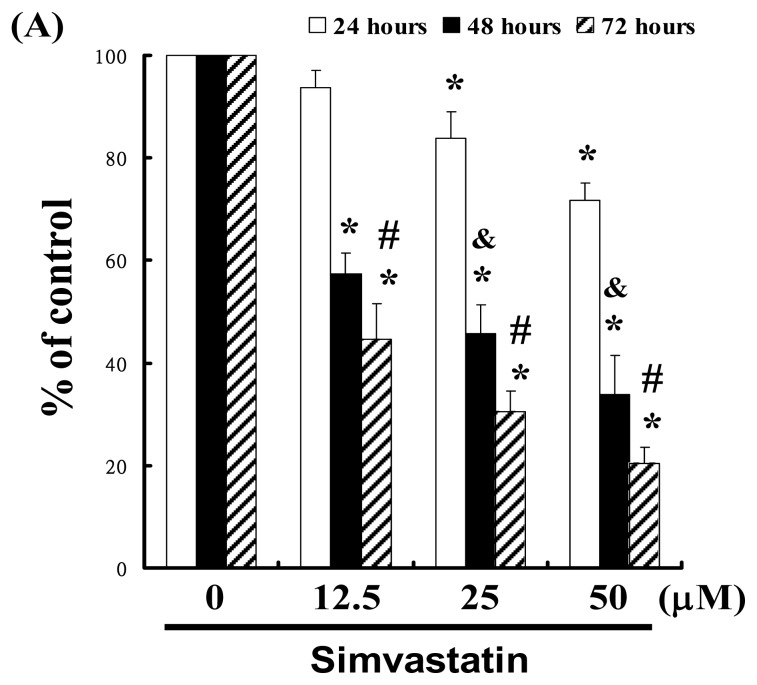

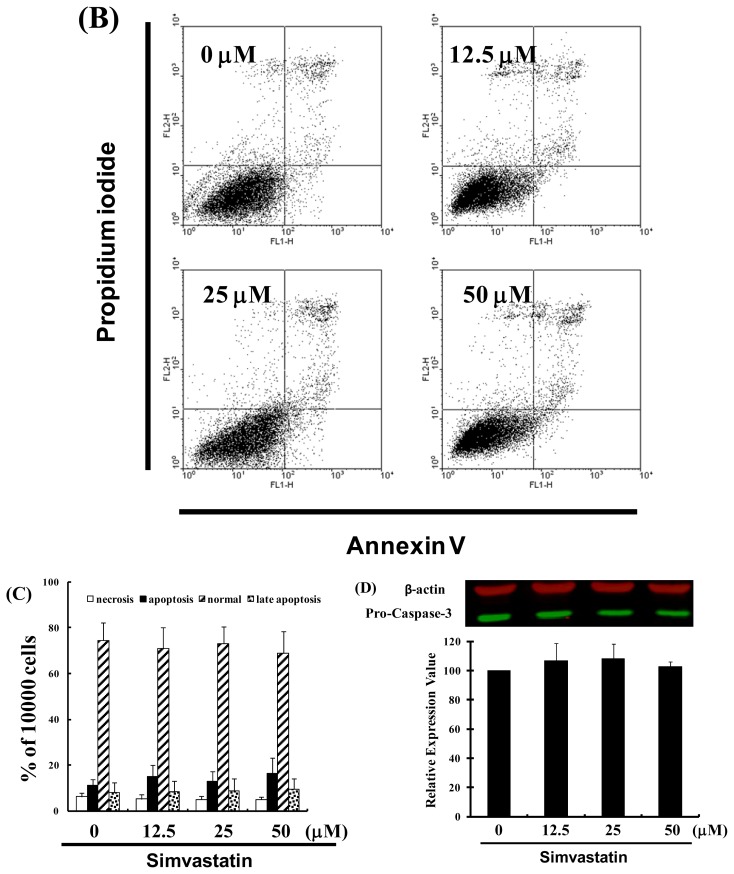
Simvastatin (SIM) mediated the survival of NCI-H460 cells, thereby inhibiting proliferation: (**A**) *In vitro* study involved the treatment of NCI-H460 cells with increasing doses of SIM for 24 to 72 h. The survival of the SIM-treated cancer cells was then determined using an MTT assay; (**B**) Influence of SIM on apoptosis in NCI-H460 cells; (**C**) Total apoptosis in NCI-H460 cells following 4 hours of incubation with SIM; (**D**) Caspase-3 activation in NCI-H460 cells induced by SIM treatment. Cells were treated with SIM, and proteins were subsequently analyzed via Western blot analysis. Band intensities (pro-caspase 3) were quantified using a Li-COR near infrared imaging system. Statistical analysis included the *t*-test. Symbols (*, & and #) in each group of bars indicate that the difference resulting from treatment with SIM is statistically significant at *p* < 0.05.

For this study, we hypothesized that SIM could mediate the survival of NCI-H460 cells by inhibiting growth. To explore the anti-tumor activity of SIM against NCI-H460 cells, we performed an *in vitro* cell viability study in which NCI-H460 cell samples were respectively treated with increasing doses of SIM (0, 12.5, 25, and 50 μM) for 24 to 72 h. The results in Figure 1A indicate that SIM treatment impaired the survival and proliferation of NCI-H460 cells in a dose-dependent manner. The 24 h IC_50_ of Simvastatin in the NCI-H460 cancer cells was determined to be 86.09 μM; *y* = −1.7209*x* + 172.14, *R*^2^ = 0.9899 (* *p* < 0.05 *vs.* SIM 0 μM group) in a time-course experiment (& *p* < 0.05 *vs.* 24 h treatment, # *p* < 0.05 *vs.* 48 h treatment). The cell viability was assayed by trypan blue exclusion and cell counting (Data not shown).

### 2.2. SIM Impaired the Viability of NCI-H460 Cells without Inducing Apoptosis

We also conducted a study on apoptosis to elucidate the anti-cancer mechanism associated with SIM in NCI-H460 cells. After treating the cells with various doses of SIM, the percentage of apoptotic cells was assessed using Annexin V-FITC and propidium iodide staining, followed by flow cytometry (Figure 1B). A dot-plot of Annexin V-FITC fluorescence *versus* PI fluorescence indicated a non-significant increase in the percentage of apoptotic cells after treatment with SIM. At SIM concentrations of 12.5 to 50 μM, no significant increase was observed in the percentage of cells undergoing necrosis or apoptosis (Figure 1C) or caspase-3 activation (Figure 1D). The results summarized in Figure 1 indicate that SIM could mediate the survival of NCI-H460 cells and, thus, inhibit their proliferation not via non-apoptotic pathways.

### 2.3. SIM Treatment Induced Accumulation of G_1_ Phase in NCI-H460 Cells

**Figure 2:**
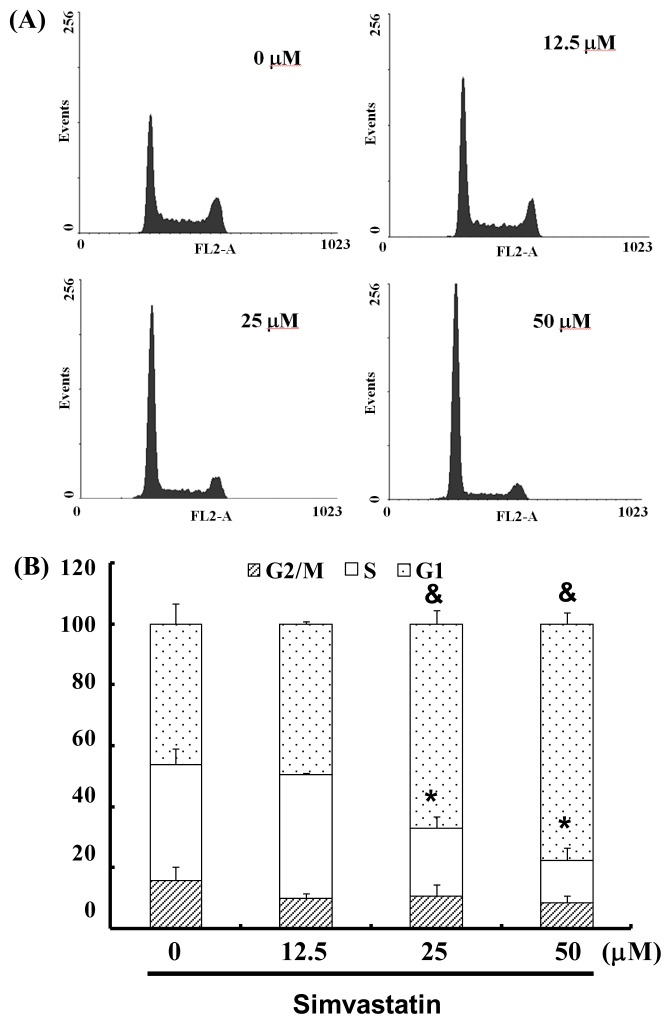

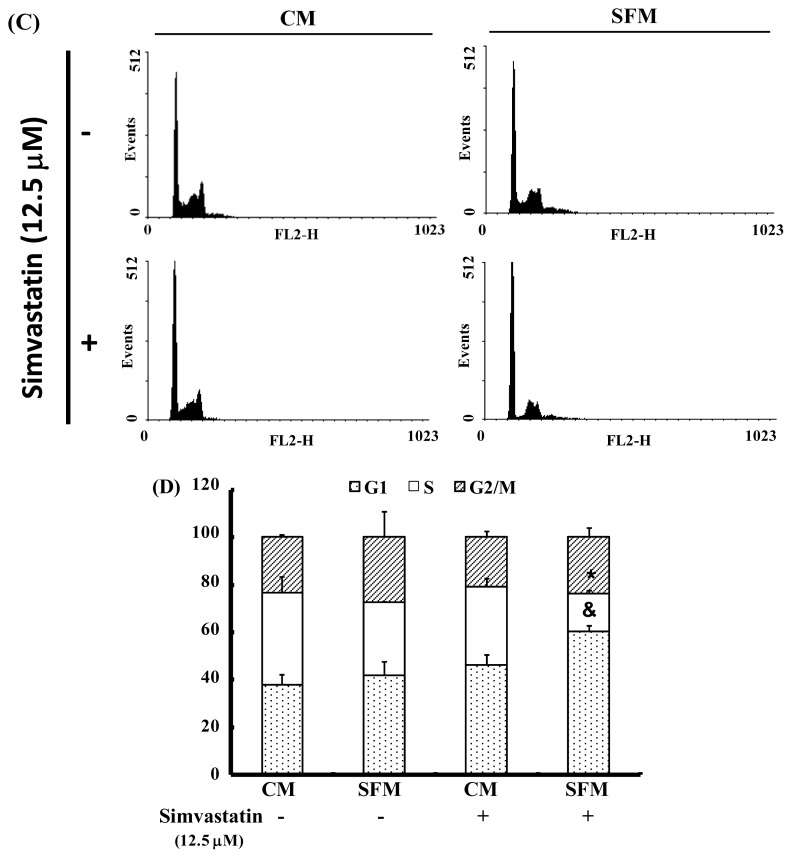
Influence of SIM on cell cycle progression/distribution in NCI-H460 cells: (**A**) Cell cycle analysis of NCI-H460 cells after being cultured with SIM for 24 h; (**B**) SIM induced an increase in the number of cells in the G_1_ phase (%) of the cell cycle. (**C**) Cell cycle analysis of NCI-H460 cells after being cultured in complete medium (CM) or serum free medium (SFM) with SIM 12.5 μM. (**D**) SIM and SFM induced an increase in the number of cells in G_1_ phase (%). Symbols (* and &) in each group of bars indicates that the differences resulting from SIM treatment is statistically significant at *p* < 0.05.

The cell-cycle distribution of SIM-treated NCI-H460 cells was analyzed using flow cytometry. Prior to processing and analysis, cells were exposed to SIM for 24 h. As illustrated in Figure 2A, the exposed cells showed an increase in the population of G1 phase cells, compared with untreated cells. These observations imply that the NCI-H460 cells may have undergone cell cycle arrest. Treatment with SIM increased the number of G_1_ phase cells (& *p* < 0.05 *vs*. SIM 0 μM) and simultaneously reduced the number of S phase cells (* *p* < 0.05 *vs*. SIM 0 μM) (Figure 2B). To investigate the role of SIM in G1 cell cycle arrest, we employed a serum deprivation (serum free) procedure to synchronize NCI-H460 cells. We chose this method due to its simplicity, reliability and reversibility. The addition of SIM increased the number of cells in G_1_ phase (& *p* < 0.05 *vs.* SIM 0 μM) and simultaneously reduced the number of cells in S phase (* *p* < 0.05 *vs.* SIM 0 μM) in CM (complete medium) and SFM (serum free medium), as shown in Figure 2C,D, respectively.

### 2.4. Cell Cycle Arrest Was Induced by SIM in NCI-H460 Cells via Cyclin D and Cdk4 Downregulation and Enhanced CDK Inhibition

**Figure 3:**
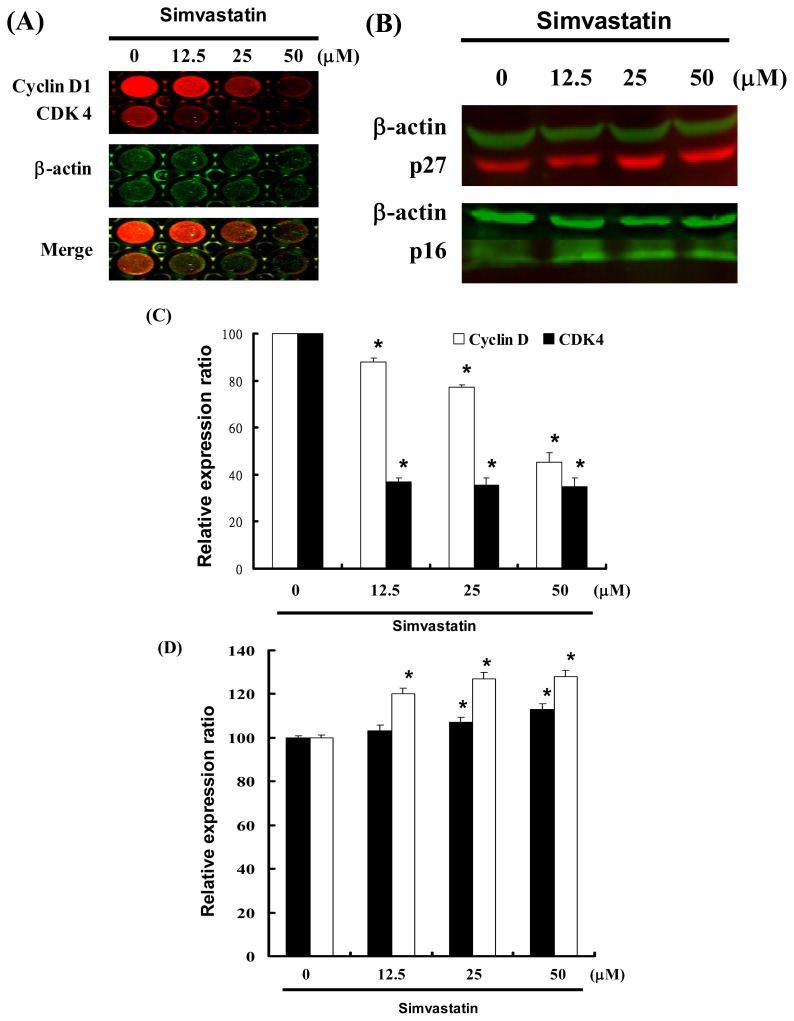
Cell cycle arrest by SIM in NCI-H460 cells via inhibition of Cdk4 and Cyclin D, in which the effects of CDK inhibitors p16 and p27 were enhanced: Cells were treated with SIM followed by (**A**) In-cell Western blot analysis (**B**) Western blot analysis (**C** and **D**) Quantification of band intensities by Li-COR near infrared imaging system. Significant differences were determined at a level of * *p* < 0.05 *versus* the 0 μM control group.

Figure 3 illustrates a protein immunoblot analysis of SIM treated NCI-H460 cells. Cyclin D and Cdk4 protein expression was quantified by measuring the relative band intensities. Results revealed a decrease in Cyclin D and Cdk4 following incubation with SIM (Figure 3A). The CDK inhibitors, p16 and p27, were also quantified by measuring the relative band intensities (Figure 3B). Cyclin D and Cdk4 levels were significantly lower in cells incubated with SIM (Figure 3C). Results show that p16 and p27 levels were significantly upregulated in cells incubated with SIM (Figure 3D).

These results indicate that SIM treatment increased the number of cells in the G_1_ phase via a downregulation of Cyclin D and Cdk4, as well as through the over expression of CDK inhibitors (p16 and p27).

HMG-CoA reductase inhibitors have demonstrated the ability to reduce albuminuria among patients with diabetes mellitus [15], transform growth factor-β1 (TGF-β1) expression [16], improve wound healing [17] and enhance endothelial function [18]. SIM is an HMG-CoA reductase inhibitor with a lipophilic compound that may provide more potent effects, such as chemo-prevention or chemo-therapy in extra-hepatic sites [19].

Cell cycle progression is regulated by CDKs and cyclin-dependent kinase inhibitors, the activity of which is largely controlled by their association with cyclins [20]. Analyses of the most common malignancies in humans have revealed mutations in these cell cycle regulators [21].

## 3. Materials and Methods

### 3.1. Materials

Simvastatin[1*S*,3*R*,7*S*,8*S*,8a*R*)-8-{2-[(2*R*,4*R*)-4-hydroxy-6-oxotetrahydro-2*H*-pyran-2-yl]ethyl}-3,7- dimethyl-1,2,3,7,8,8a-hexahydronaphthalen-1-yl 2,2-dimethylbutanoate] was obtained from Sigma (St. Louis, MO, USA). RPMI 1640 medium, fetal bovine serum, antibiotics, trypsin and phosphate-buffered saline (PBS) were purchased from Gibco, BRL (Grand Island, NY, USA). All other reagents and compounds were analytical grade.

### 3.2. Cell Culture

NCI-H460 cells were purchased from the Food Industry Research and Development Institute (Hsinchu, Taiwan). The cells were maintained on culture dishes, in RPMI 1640 medium supplemented with 10% (*v*/*v*) FBS and cultured in an incubator at 37 °C in an atmosphere containing 5% CO_2_.

### 3.3. Cell Proliferation Assay

Cells were seeded into 96-well culture plates at 10,000 cells/well and treated with SIM for 1–3 days. MTT dye (1 mg/mL) was added for an additional 4 h following treatment. The reaction was stopped by the addition of DMSO, whereupon OD_540_ was measured using a multi-well plate reader (Powerwave XS, Biotek, Seattle, WA, USA). In the absence of cells, the background absorbance of the medium was subtracted. The cell viability assays were determined by trypan blue analysis. Results were expressed as a percentage of the control, which was considered 100%. Each assay was performed in triplicate, and the results were expressed as the mean (±SEM).

### 3.4. Measurement of Apoptosis

NCI-H460 cells were first seeded in 6-well plates (Orange Scientific, E.U.). The cells were harvested after treatment with SIM for 4 h. The cells were then re-centrifuged (the supernatant was discarded) and resuspended/incubated for 15 min in 1× annexin-binding buffer, 5 μL of FITC annexin V (BD Pharmingen, San Jose, CA, USA) and 1 μL of 100 μg/mL PI working solution. Following incubation, the stained cells were analyzed using flow cytometry (FACSCalibur, San Jose, CA, USA). Data was analyzed using WinMDI 2.8 free software (BD, San Jose, CA, USA).

### 3.5. Cell Cycle Analysis

For cell cycle analysis, we employed the fluorescent nucleic acid dye, propidium iodide (PI), to identify the proportion of cells in each of the three interphase stages of the cell cycle. Cells were treated with SIM for 24 h and then harvested and fixed in 1 mL cold 70% ethanol for at least 8 h at −20 °C. DNA was stained in PI/RNaseA solution, and the DNA content was detected using flow cytometry. Data was analyzed using WinMDI 2.8 free software (BD, San Jose, CA, USA).

### 3.6. Western Blot Assay

A total of 30–50 μg of proteins were separated using 12% SDS-PAGE and transferred to PVDF membranes (Millipore, Billerica, MA, USA). The membranes were blocked with blocking buffer (Odyssey, Englewood, NJ, USA) overnight and subsequently incubated with anti-β-actin (Sigma-Aldrich, St. Louis, MO, USA), anti-p16 (Abcam, Cambridge, UK), anti-caspase-3 and anti-p27 (Santa Cruz BioTechnology, Santa Cruz, CA, USA) antibodies for 1.5–2 h. The blots were then washed and incubated with a secondary antibody (IRDye Li-COR, Lincoln, NE, USA) at a dilution of 1/20,000 for 30 min. The antigen was then visualized using a near infrared imaging system (Odyssey LI-COR, Lincoln, NE, USA) and data was analyzed using Odyssey 2.1 software.

In-cell Western blotting is an effective means to quantify intracellular signaling in whole cells. Briefly, this assay process involves seeding cells in microtiter plates, followed by fixation/permeabilization and subsequent labeling using anti-Cyclin D (Santa Cruz BioTechnology, Santa Cruz, CA, USA), Cdk4 (Abcam, Cambridge, UK), β-actin (Sigma-Aldrich, St. Louis, MO, USA) and infrared-conjugated secondary antibodies. The Odyssey near infrared imaging system obtains well-level data.

### 3.7. Statistical Analysis

All data was reported as the mean (±SEM) of at least three separate experiments. A *t*-test or one-way ANOVA with post-hoc test was employed for statistical analysis, with significant differences determined as *p* < 0.05.

## 4. Conclusions

In the current study, SIM induced G1 cell cycle arrest in NCI-H460 cells, indicating the existence of an additional mechanism by which SIM may inhibit the proliferation of NSCLC cells. To analyze the biological mechanisms underlying SIM-induced G1 cell cycle arrest, we evaluated the protein and gene expressions of CDKs and cyclins, as well as CDK-inhibitors p16 and p27. The SIM-induced reduction of the number of cells in S phase and G_1_ cell cycle arrest was associated with a marked reduction in Cyclin D-Cdk4 protein levels. Our results suggest that SIM-induced G_1_ cell cycle arrest in NCI-H460 cells is mediated by the upregulation of p16 and p27 proteins, which in turn indicates that the cell cycle-suppressing activity of SIM is associated with its anti-cancer properties.
